# A dried blood spot protocol for high-throughput semi-quantitative analysis of Epstein-Barr Virus VCA IgG and EBNA IgG serologies based on the Roche Elecsys system

**DOI:** 10.1186/s12985-026-03219-w

**Published:** 2026-07-23

**Authors:** Raquel Rubio-Acero, Christina Reinkemeyer, Mona Niethammer, Melina Hufnagl, Kalina Kazandzhieva, Irene Charlotte Schoof, Simon Winter, Alberto Beyersdorff Filho, Jessica Hintze, Thomas Seeholzer, Jana-Kristin Heise, Stefanie Castell, Thomas P. H. Meyer, Mirjam Schunk, Michael Hoelscher, Noemi Castelletti, Andreas Wieser

**Affiliations:** 1 Institute of Infectious Diseases and Tropical Medicine, LMU University Hospital, LMU Munich, 80802 Munich, Germany; 2 Fraunhofer Institute for Translational Medicine and Pharmacology ITMP, Immunology, Infection and Pandemic Research IIP, Türkenstraße 87, 80799 Munich, Germany; 3 Max-von-Pettenkofer Institute, LMU Munich, Munich, Germany; 4 Department of Epidemiology, Helmholtz Centre for Infection Research, Braunschweig, Germany; 5 German Center for Infection Research (DZIF), partner site Munich, Munich, Germany; 6 Center for International Health (CIH), University Hospital, LMU Munich, 80336 Munich, Germany; 7 Unit Global Health, Helmholtz Zentrum München, German Research Centre for Environmental Health (HMGU), Neuherberg, Germany; 8 Institute of Medical Biostatistics, Epidemiology and Informatics (IMBEI), University Medical Center, Johannes Gutenberg University, 55101 Mainz, Germany

**Keywords:** EBV, Infectious Mononucleosis, Dried Blood Spot, DBS, Filter paper, Antibody, Serology, Roche Elecsys, EBNA-1, VCA

## Abstract

**Background:**

Epstein-Barr virus (EBV) is highly prevalent worldwide and has been linked to different cancers and autoimmune disorders, including Multiple sclerosis (MS). Primary infection typically occurs during early childhood or adolescence and often goes undetected. Serological studies allow the detection of EBV-specific antibodies after infection, but venous blood collection is resource-intense and thus limits sample size. To overcome these challenges, we developed a high-throughput, semi-automated serology protocol targeting antibodies against EBV in capillary blood samples (Dried Blood Spots, DBS).

**Methods:**

Paired serum and DBS samples were obtained from 417 participants aged 18–25 years. We used Roche Elecsys^®^ EBV nuclear antigen-1 EBNA IgG and Elecsys^®^ EBV viral capsid antigen VCA IgG assays, established for serum, to detect antibodies against EBV anti-EBNA‑1 IgG and anti-VCA IgG in DBS. Cut-off Indices (COI) for the DBS assays were determined using a classification tree.

**Results:**

Of the 416 valid paired serum samples, 78.6% (327/416) were anti-VCA-positive and 76.4% (318/416) anti-EBNA-positive. Discrepant results were observed in 3.6% (15/416) of participants. Based on combined assay results, 20.7% (86/416) of the samples were classified as EBV-negative. The serum COI values revealed that most of the negative cases clustered well below the cutoff, while positive cases spanned a broad range of higher values. Overall, 98.8% (326/330) of participants classified as positive based on serum testing (*n* = 330) were also classified as positive in DBS (*n* = 326), corresponding to the sensitivity of DBS relative to serum results. Likewise, 96.5% (83/86) of participants classified as serum-negative participants (*n* = 86) were also negative in DBS (*n* = 83), corresponding to the specificity of DBS relative to serum results. Among the 416 participants, only 4/330 (1.2%) were false negatives and 3/86 (3.5%) were false positives in DBS. The newly established assays were validated in a self-sampling cohort of 295 participants.

**Conclusion:**

We established DBS-specific cutoff values to detect antibodies against EBV in DBS, achieving high sensitivity (98.8%) and specificity (96.5%) relative to the corresponding venous blood assay. Furthermore, we derived a correction formula to convert semi-quantitative DBS values to serum-equivalents, enabling comparison with other studies and standardized datasets. The DBS-based approach allows sero-status assessment in a large population and thus e.g. simplifies the identification of suitable participants for clinical trials.

**Supplementary Information:**

The online version contains supplementary material available at 10.1186/s12985-026-03219-w.

## Introduction

The Epstein-Barr Virus (EBV) is a human gammaherpesvirus with strong B-lymphotropism, lifelong latency and high worldwide prevalence. Global seropositivity in adults is estimated to be above 95% [1, 2]. While primarily transmitted via saliva, alternative transmission routes such as breast milk, bodily fluids, and organ transplantation have also been documented [1]. Among adolescents and young adults, intimate oral contact is a major transmission mode [3].

Primary infection typically occurs during early childhood or adolescence. Infections at a younger age are often asymptomatic or resemble mild respiratory illness and therefore frequently go undetected. In contrast, infections acquired later in life tend to be more severe and may lead to long-term health consequences [2].

First identified in 1964 in patients with Burkitt lymphoma, EBV has since been linked to several other lymphoproliferative diseases, including Hodgkin lymphoma (HL), non-HL in post-transplant patients and HIV-infected individuals, T-cell lymphoma, and natural killer/T-cell lymphoma [1, 2]. Beyond lymphomas, EBV is causally associated with nasopharyngeal carcinoma [2] and contributes to approximately 10% of gastric cancer cases globally [1, 2]. Moreover, EBV has been linked to various autoimmune disorders, including rheumatoid arthritis, Sjögren’s syndrome, systemic lupus erythematosus, and Multiple Sclerosis (MS) [1, 2, 4]. The timing of EBV infection appears to be critical: while infections in early childhood can cause cancer later in life, individuals infected later in life present a higher risk of MS [5, 6].

Symptomatic primary EBV infection manifests as infectious mononucleosis (IM), a condition marked by pharyngitis, cervical lymphadenopathy, fatigue, and fever. IM can lead to significant morbidity and has been associated with long-term complications such as severe myalgic encephalomyelitis/chronic fatigue syndrome and serves as a known risk factor for the development of HL and MS [1, 3].

Following infection, the immune system produces antibodies against viral capsid antigens (VCA), with anti-VCA IgM and IgA appearing early and persisting for several weeks to months. In contrast, anti-VCA IgG normally remains detectable for life [2]. Another key antigen, EBV nuclear antigen-1 (EBNA-1), is the target for anti-EBNA-1 antibodies typically appearing around three months post-infection. Anti-EBNA-1 IgG antibodies, like anti-VCA IgG, persist long-term and are considered reliable indicators of past EBV infection [3].

The wide range of EBV-associated diseases has spurred interest in the development of vaccines to prevent infection and mitigate severe outcomes. However, to date, no licensed vaccines or targeted therapies exist for IM or its long-term complications [2, 3].

However, it is essential to identify EBV-seronegative individuals for vaccine development. The high global prevalence of EBV and the young age at which primary infection occurs present significant challenges in recruiting sufficient numbers of EBV-negative participants for clinical trials. Even conventional high-throughput serological testing, which typically relies on venous blood, leads to considerable costs due to the necessary appointments, resource-intensive processes, and the need for trained medical personnel to obtain the samples. Therefore, even in well-developed healthcare systems, it remains challenging to conduct consistent, large-scale sero-epidemiological cohorts and eventually recruit seronegative subjects for early-stage vaccine trials.

In order to avoid the time-consuming and cost-intensive conventional blood collection, a promising alternative to venous blood sampling is the use of self-sampling techniques such as dried blood spots (DBS) on filter paper [7, 8]. DBS are acquired by applying a small amount of capillary blood obtained from a finger prick with a lancet onto a filter paper and allowing it to dry before transportation (7). This procedure offers several advantages: (i) it is only minimally invasive and thus does not require trained medical personnel, and (ii) DBS cards are small and once dried, are easy to transport and store for extended periods of time [7, 9–11].

Although commercial assays for detecting EBV-specific antibodies are well established for serum, adapting these for DBS requires tailored laboratory protocols for sample extraction and processing.

McDade et al. [12] and Eick et al. [8], successfully implemented DBS-eluate based serology testing for EBV antibodies using Enzyme-linked immunosorbent assay (ELISA) format. However, chemiluminescence immunoassay (CLIA) techniques have shown to be more sensitive, specific, and rapid [13]. In this study, we aim to develop a high-throughput, semi-automated DBS-based serology protocol targeting anti-EBNA-1 IgG and anti-VCA IgG antibodies, using the Roche electrochemiluminescence (ELECSYS) format. We used paired DBS and serum samples from participants aged 18–25 years in order to (i) identify relevant cut-off values by DBS for the detection of EBV antibodies, (ii) compare the test performance of DBS to conventional blood serum assay, (iii) test the assay in a self-sampled cohort, and (iv) derive a correction formular to convert semi-quantitative DBS results to serum-equivalents, enabling comparison with other studies and standardized datasets.

## Materials and methods

### Participant samples

To establish and validate the serological assays, paired serum and DBS samples (i.e., serum and DBS collected from the same individual in parallel) were obtained from 417 participants aged 18–25 years. Recruitment took place from November 2023 to February 2024 via leaflets, posters, and digital advertisements in medical and university settings in Munich, Germany. Exclusion criteria included any pre-existing diseases or conditions that impaired the ability to provide informed consent, as well as insufficient proficiency in the German language that could hinder comprehension of the consent process. Participants were invited to the clinical trial unit of the Institute of Infectious Diseases and Tropical Medicine at LMU University Hospital, where trained medical personnel collected the paired samples.

DBS samples were obtained from capillary blood using single-use safety lancets (Sarstedt 85.1016). Blood was collected by medical staff on filter paper cards (8.460.0004.A Rev.1, Ahlstrom-Munksjö), filling up to five circular collection areas. After blood collection, DBS were left to air-dry for 12–24 h at room temperature protected from direct sunlight and analyzed within 48–72 h. Following analysis, DBS samples were stored dry at − 80 °C in zip-lock bags containing desiccant packs.

To study the deterioration of titre values over time, a subset of DBS samples was subjected to different conditions: (i) freezer (− 18 °C to − 21 °C, laboratory storage), (ii) refrigerator (4 °C to 7 °C, simulating winter shipment), (iii) room temperature (22 °C to 25 °C, short term home storage/drying and shipment in spring/fall), (iv) incubator (36 °C to 38 °C, simulating elevated temperatures during mailing), (v) direct sunlight (exposure to full-day sunlight on a window ledge at room temperature, simulating incorrect drying), and (vi) wet packaging (less than 3 h of drying before packaging). Each temperature condition was applied for 11 consecutive days post-collection to account for potential delays in mailing.

Paired venous blood samples were collected in 2.7 mL S-Monovette^®^ Serum CAT tubes (Sarstedt. 04.1943.001). Serum was obtained following manufacturer recommendations [14]. Aliquots were stored at -80 °C in temperature-controlled biobank freezers.

### Validation of sampling methods

DBS sample self-collection may generate handling challenges, such as insufficient blood volume, resulting in impossibility to workup the sample or other problems [10]. To assess the feasibility of the assay in self-collected samples, a sub study was conducted in a newly recruited population fulfilling the same inclusion criteria as the previous group.

Recruitment took place between May to September 2024 via leaflets, posters, and digital advertisement in the same medical and university setting as described above. Individuals were recruited remotely, using the Prospective Monitoring and Management Application (PIA). PIA is an open-source eResearch system developed for the management of observational epidemiological studies [15, 16]. It allows interested individuals to self-register and provide Informed Consent to study activities by filling in a digital form. Upon self-enrolment, eligibility and quality checks were performed by the study team who subsequently included participants into the study. Participants received blood collection kits (Euroimmun ZV 9702–0101) by mail and were instructed to perform the DBS collection independently at home. Detailed written instructions and a link to a video tutorial [17] were provided to guide them through the process. Subsequently, the newly developed DBS assays were validated on 295 self-sampled DBS.

### DBS quality control of self-collected DBS

For self-collection of DBS samples, participants were instructed to fill up at least two of the five circular collection areas on the filter cards and allow the DBS to dry for a minimum of 12 h at room temperature, protected from heat and direct sunlight. Participants were then asked to return the DBS cards within three days, sealed in a zip-lock bag containing a desiccant sachet. Upon receipt at the laboratory, the DBS cards were registered by scanning their barcodes into a database and subsequently stored at -80 °C in temperature-controlled biobank freezers until analysis.

### Extraction of antibodies from the DBS

To achieve a high sample throughput to detect anti-EBNA‑1 IgG and anti-VCA IgG antibodies in DBS, we optimized the semi-automated workflow reported elsewhere [9, 10]. In brief, previously tempered DBS samples were punched with a Panthera-Puncher 9 Instrument (PerkinElmer). A total of three discs with 3.2 mm in diameter per DBS sample were automatically assigned by the machine to be punched if not indicated otherwise. Discs were collected in a barcoded 96-well plate. 80 µL of elution buffer per well was dispensed with adjustable tip spacing multichannel pipettes (Integra Voyager), and elution was performed in a temperature-controlled shaker (MIUlab ES-60E) for 1 h at 37 °C and 300 rpm. Roche 13/16 micro sample cups (Roche, 05085713001) were used to minimize the dead-volume needed for analysis in the Roche Elecsys system. DBS samples were stored at -80 °C in temperature-controlled biobank freezers after analysis. During freezing and thawing, DBS cards were protected from condensation and humidity by using zip-lock bags and desiccant sachets.

### Roche immunoassay

The Roche Elecsys^®^ EBV EBNA IgG and Elecsys^®^ EBV VCA IgG assays are commercially available in vitro diagnostic (IVD) immunoassays validated for the qualitative detection of EBV-specific IgG antibodies in human serum and plasma according to the manufacturer’s specifications [18]. The assays use recombinant proteins representing EBNA-1-specific and VCA-specific antigens in a double-antigen sandwich assay format, detecting IgG antibodies against nuclear antigen-1 (EBNA-1) and viral capsid antigens (VCA) respectively. The immunoassay was performed on a Cobas e801 analytical unit (Roche) using electrochemiluminescence (ELECSYS) technology. The results are given as Cut-Off Index (COI). In the present study, serum samples analyzed with these validated IVD assays were considered as the reference standard for evaluation of DBS performance. To ensure comparability between matrices, paired DBS and venous blood samples from each participant were analyzed on the same day using the same reagent lot and analytical platform. In addition, an extensive analytical validation was performed to assess the applicability of the assays to DBS eluates and to evaluate the use of raw COI values for (semi-) quantitative interpretation of antibody responses beyond the manufacturer-defined qualitative classification.

### Data handling

Data handling followed the same procedures as described in previous studies [9, 10]. Each DBS card was assigned a unique barcode, which was automatically scanned during the punching process. The punching device automatically recorded the linkage between the DBS barcode and its corresponding position on the 96-well plate, into which paper discs were dispensed. Each 96-well plate was labeled with a unique barcode to ensure unambiguous identification. To prevent processing errors, the punching software was configured to block repeated punching of the same barcoded filter paper within a single plate. Sample and barcode information were transferred to the Janus robotic system via a custom-developed script. Eluates were subsequently loaded into micro sample cup inserts, arranged in barcoded rack packs in batches of five samples. In parallel, a corresponding worklist was generated for the Cobas analyzer and transferred automatically, enabling both automated sample analysis and export of results, including luminescence signal values, directly linked to the respective DBS sample barcode.

### Statistics

Prior to statistical analysis, the data was cleaned and locked. Participants were considered seropositive if at least one of the two assays yielded a positive result. All analyses and visualizations were performed using the R software, version 4.4.2.

To ensure that assay measurements fell within the linear detection range, both DBS and serum samples were diluted using matrix-specific thresholds based on their respective raw COI values. Due to the inherent dilution introduced during DBS extraction, DBS eluates yield lower COI values than serum for comparable antibody levels and therefore require different cut-offs for additional dilution. For DBS eluates, samples with values ≥ 10 COI for anti-EBNA-1 antibodies and ≥ 80 COI for anti-VCA antibodies were diluted 1:10. Serum samples with anti-VCA values ≥ 1500 COI or anti-EBNA values ≥ 130 COI were diluted 1:20. The true concentrations were then obtained by back-calculating the respective dilution factors.

Cutoff values for anti-EBNA antibodies and anti-VCA antibodies were empirically determined as interdependent, using all valid paired DBS and serum samples. To identify the optimal cutoffs for DBS samples, a classification tree was fitted using serum-based anti-EBNA and anti-VCA results as the ground truth. At each node of the classification tree, the algorithm minimized the Gini index, a measure of node impurity that reflects the degree to which seropositive and seronegative cases (based on serum data) are mixed. A node is considered pure when all observations belong to the same class. For each split, the model selected either DBS-based antibodies against EBNA or VCA as the predictor variable and determined the cutoff that maximally reduced node impurity, thereby improving the accuracy of seroprevalence classification [19]. Each node in the tree displays four decimal values representing the proportion of individuals falling into the four serum-based EBNA/VCA classification categories: (i) double negative, (ii) anti-EBNA negative/anti-VCA positive, (iii) anti-EBNA positive/anti-VCA-negative, and (iv) double positive. These proportions illustrate how DBS-based classifications align with the reference serum-based categories and highlight potential misclassifications. The label assigned to each node corresponds to the most frequent serum-based classification in that group, i.e., the category with the highest proportion among the four.

Global sensitivity and specificity of the DBS-based cutoffs were calculated with reference to the serum results, representing the proportions of samples correctly classified as positive or negative in both specimen types.

Associations between continuous variables were assessed using Spearman’s rank correlation coefficient. In addition, Bland–Altman plots were generated for both assays to assess the agreement between serum and DBS values transformed to serum-equivalent units.

For the analysis of decay characteristics of DBS, three different donors produced multiple DBS cards, and time series analyses were performed as individual triplicates over time. The difference to the baseline value was calculated by computing (i) the mean per participant and per assay by baseline and (ii) the difference in percentage per participant per assay for all the timepoints. The Wilcoxon test was applied to assess significant differences across measurement groups.

To enable conversion of DBS-derived antibody values to serum-equivalent concentrations, a correction formula was derived:

$$\begin{aligned}VenousUnits={10}^{intercept+slope\cdot\:{\mathrm{log}}_{10}DBSUnits}\end{aligned}$$

This formula was established using the ordinary nonparametric bootstrap method with 10,000 bootstrap replicates, providing mean estimates and bias-corrected and accelerated (BCa) 95% confidence intervals (CI) for both the slope and intercept [20, 21, 22].

## Results

**Fig. 1:**
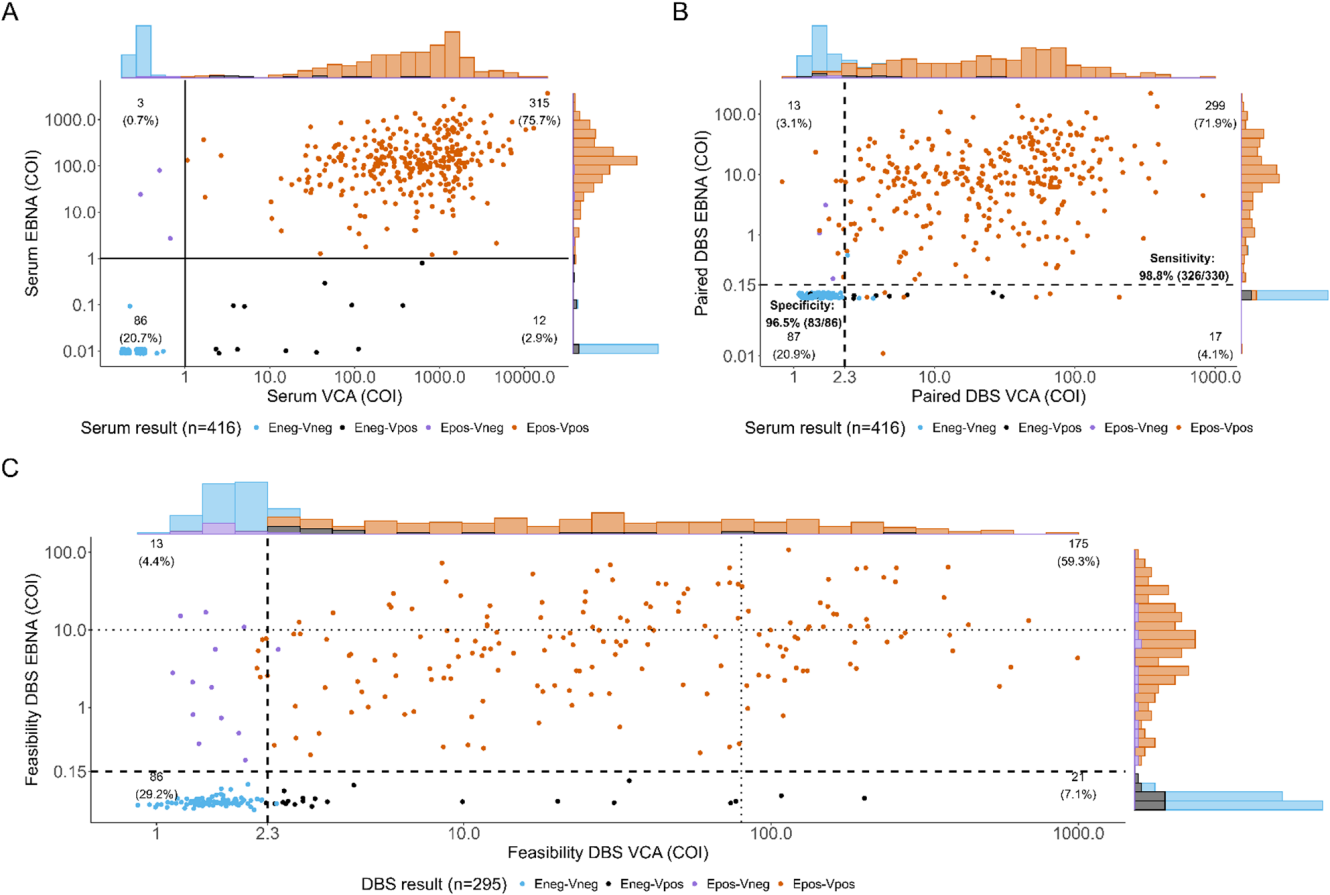
Scatterplots with marginal distributions showing the relationship between anti-EBNA and anti-VCA results. Solid lines represent the manufacturer’s serum cutoff (1 COI) for result classification. Dashed lines indicate empirically determined DBS cutoffs (2.3 for anti-VCA; 0.15 for anti-EBNA), and dotted lines mark the assays’ linear rage limits (80 for anti-VCA and 10 for anti-EBNA). (**A**) Paired serum samples and (**B**) paired DBS samples collected by medical personnel (*n* = 416), including sensitivity and specificity calculations of DBS assays relative to serum. (**C**) Self-collected DBS samples from participants at home (*n* = 295). Eneg = anti-EBNA-1 antibody negative, Epos = anti-EBNA-1 antibody positive, Vneg = anti-VCA antibody negative, Vpos = anti-VCA antibody positive

Of 417 participants included, a total of 416 participants met the eligibility criteria; one participant had no measurement for serum and was therefore excluded from the analysis. Of the 416 paired serum–medical staff collected DBS samples, 78.6% (327/416) were anti-VCA-positive and 76.4% (318/416) anti-EBNA-positive (Fig. 1A). Discrepant results were observed in 3.6% (15/416) of participants: 0.7% (3/416) were anti-VCA-negative but anti-EBNA-positive, and 2.9% (12/416) were anti-VCA-positive but anti-EBNA-negative. Based on combined assay results, 20.7% (86/416) of the samples were classified as anti-EBV-negative. The serum COI values for anti-EBNA and anti-VCA showed distinct distributions, with most of the negative cases clustering well below the cutoff, while positive cases spanned a broad range of higher values.

**Fig. 2:**
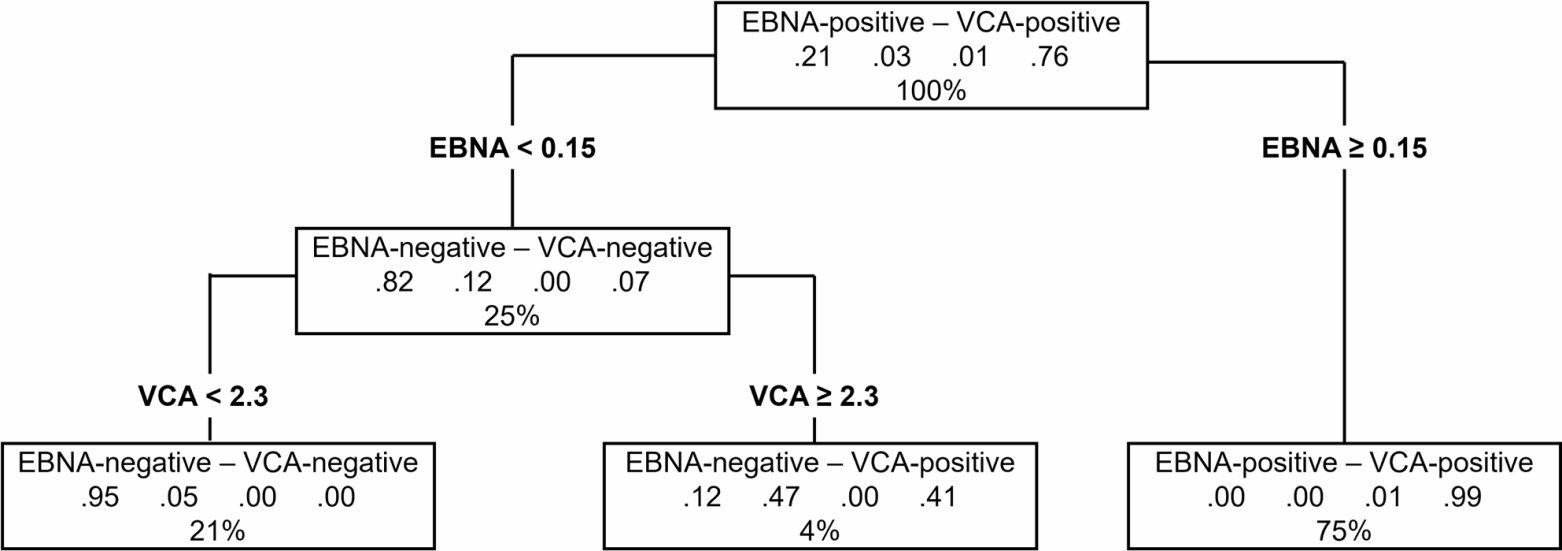
Classification tree for determining empirical, interdependent cutoffs for DBS samples (*n* = 416). The tree was fitted using the composite serum-based anti-EBNA and anti-VCA COI results as the reference standard. The root node (*n* = 416) is first split by the anti-EBNA threshold (0.15); samples with anti-EBNA COI < 0.15 are then further split by anti-VCA (threshold: 2.3). Terminal nodes show the final DBS-based classification groups and the percentage of participants in each. The four decimal values at each node represent the distribution of serum-based classifications: anti-EBNA-negative/anti-VCA-negative, anti-EBNA-negative/anti-VCA-positive, anti-EBNA-positive/anti-VCA-negative, and anti-EBNA-positive/anti-VCA-positive (in this order)

Based on these serum-based results, cutoffs for the DBS assays were determined using a classification tree (Fig. 2). The first split is based on the anti-EBNA COI values: participants with anti-EBNA COI ≥ 0.15 were directly classified as positive (75%), while those with anti-EBNA COI < 0.15 were further stratified by anti-VCA values. Anti-EBNA-negative participants with anti-VCA COI < 2.3 were classified as double negative (21%), while those with anti-VCA COI ≥ 2.3 were considered anti-EBNA-negative and anti-VCA-positive (4%). The nodes indicated the most frequent DBS-based classification in this split, with the percentage shares of all potential serum-based classification categories below.

The distributions of the DBS assay results did not exhibit the same clearly defined clustering of the negative group as observed in the serum samples (Fig. 1B). For anti-EBNA antibodies, the distribution revealed a more distinct gap, allowing for clearer separation between positive and negative samples. In contrast, the anti-VCA antibody distribution, while still having a negative interval, appeared as continuous, lacking a clearly visible gap between the populations.

Overall, 98.8% (326/330) of participants who tested positive in serum were also classified as positive in DBS, reflecting this level of sensitivity. Likewise, 96.5% (83/86) of serum-negative participants were also negative in DBS, corresponding to this level of specificity. Among the total 416 participants, only 4 (1.2%) were false negatives and 3 (3.5%) were false positives in DBS compared to the serum values of the same day.

Examining the DBS distributions of the 295 self-collected samples taken at home revealed no major differences compared to those collected by medical personnel (Fig. 1C). Using the cutoff of 0.15 for anti-EBNA antibodies, 63.7% (188/295) of the samples tested positive, while for anti-VCA antibodies, the cutoff of 2.3 yielded 66.4% (196/295) positivity. Based on combined assay results, 29.2% (86/295) of individuals were subsequently classified as EBV-negative.

**Fig. 3:**
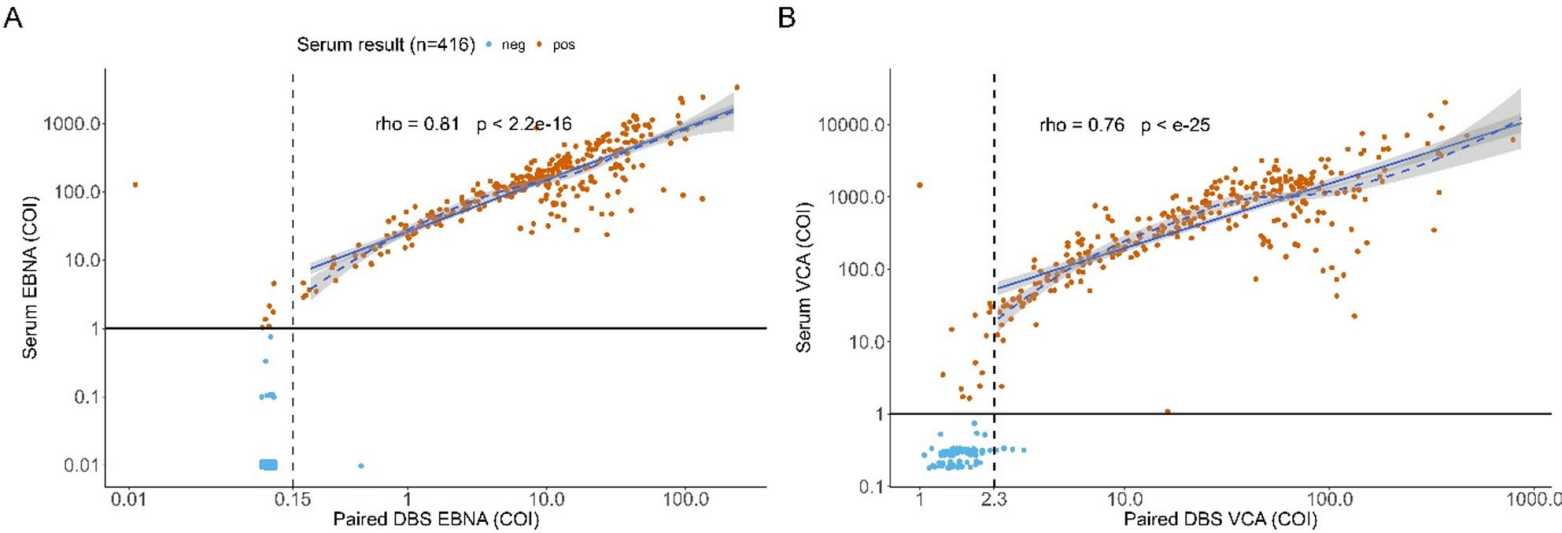
Scatterplots showing the linear log10–log10 relationship between antibody concentrations in DBS eluates (x-axis) and corresponding venous serum (y-axis) for (A) anti-EBNA antibodies and (B) anti-VCA antibodies (*n* = 416). Correlations were assessed using Spearman’s method. Solid horizontal lines indicate the manufacturer’s cutoff for serum classification (1 COI), and dashed vertical lines indicate empirically determined cutoffs for DBS classification (0.15 COI for anti-EBNA; 2.3 COI for anti-VCA). Solid blue lines represent linear regressions; dashed blue lines show LOESS fits (locally estimated scatterplot smoothing or local regression). Shaded gray regions denote 95% CI for the linear- and LOESS estimates, respectively

To evaluate the feasibility of extracting (semi-) quantitative information from DBS eluates, we analyzed all paired serum and DBS samples using the raw COI values in a semi-quantitative manner. The results were plotted on a log10/log10 scale (Fig. 3), revealing strong correlations between DBS eluates and serum values (Spearman’s rho = 0.81 for anti-EBNA and 0.76 for anti-VCA). Using ordinary nonparametric bootstrap resampling, we derived correction formulas to estimate serum-equivalent units from DBS measurements:

$$\begin{aligned}\:EBNA\:VenousUnits\\={10}^{1.408+0.767\cdot\:{\mathrm{log}}_{10}EBNA\:DBSUnits}\end{aligned}$$

$$\begin{aligned}\:VCA\:VenousUnits\\={10}^{1.400+0.892\cdot\:{\mathrm{log}}_{10}VCA\:DBSUnits}\end{aligned}$$

Adjusted bootstrap percentile (BCa) confidence intervals, calculated from 10,000 replicates, were (1.368, 1.454) for the intercept and (0.705, 0.818) for the slope in the case of anti-EBNA antibodies, and (1.277, 1.52) and (0.797, 0.979), respectively for anti-VCA antibodies.

**Fig. 4:**
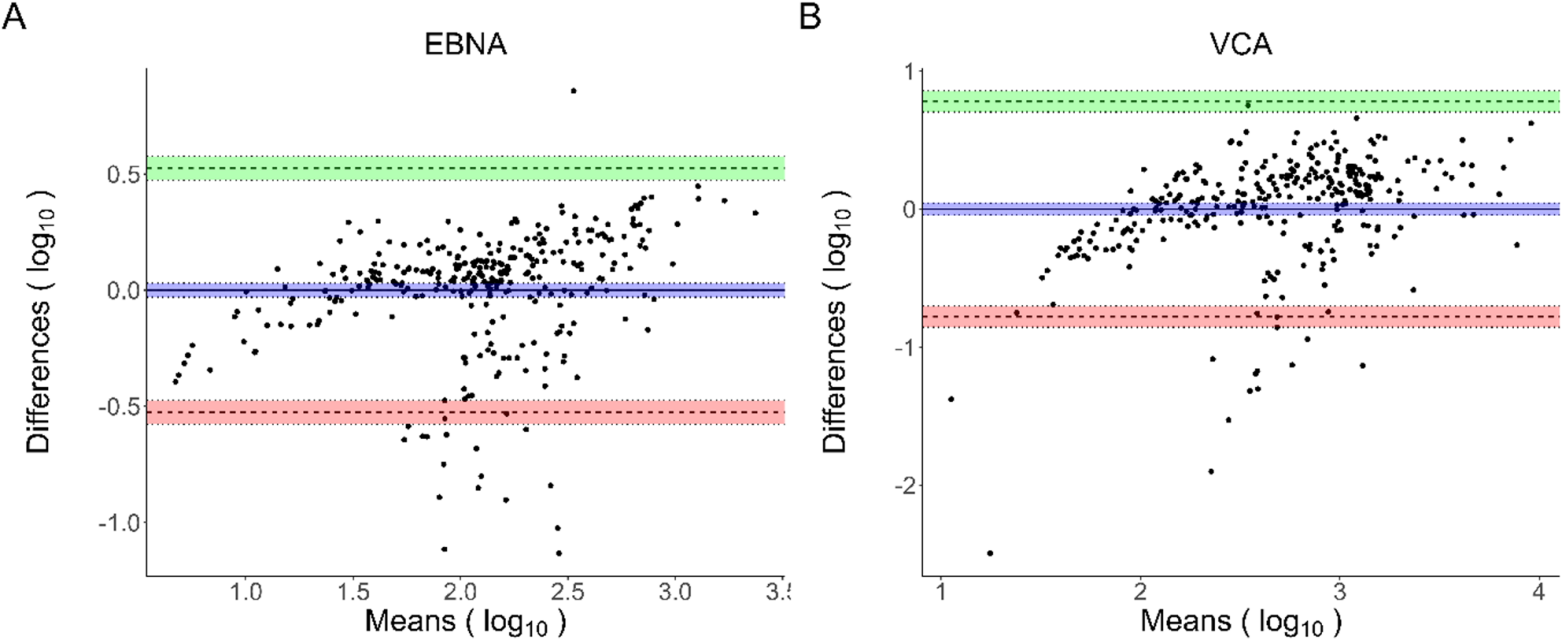
Bland-Altman plots showing the agreement between serum values and DBS values converted to serum units, with both displayed on a log10 scale. The mean signal difference between DBS converted and serum values is (**A**) anti-EBNA: $\:1.1\cdot\:{10}^{-15}$ COI, with 95% of values falling within the interval (-0.525 COI; +0.525 COI), and (**B**) anti-VCA: $\:1.7\cdot\:{10}^{-15}$ COI, with 95% of values falling within the interval (-0.779 COI; +0.779 COI)

To further assess agreement between serum and DBS values transformed to serum-equivalent units, Bland–Altman plots were generated for both assays (Fig. 4). The differences were symmetrically distributed around zero, showing no evidence of proportional bias. When back-transformed from the log10 scale, mean differences were negligible: $\:1.1\cdot\:{10}^{-15}$ COI for Anti-EBNA (95% interval: -0.525—+0.525 COI) and $\:1.7\cdot\:{10}^{-15}$ COI for anti-VCA (95% interval: -0.779—+0.779 COI).

**Fig. 5:**
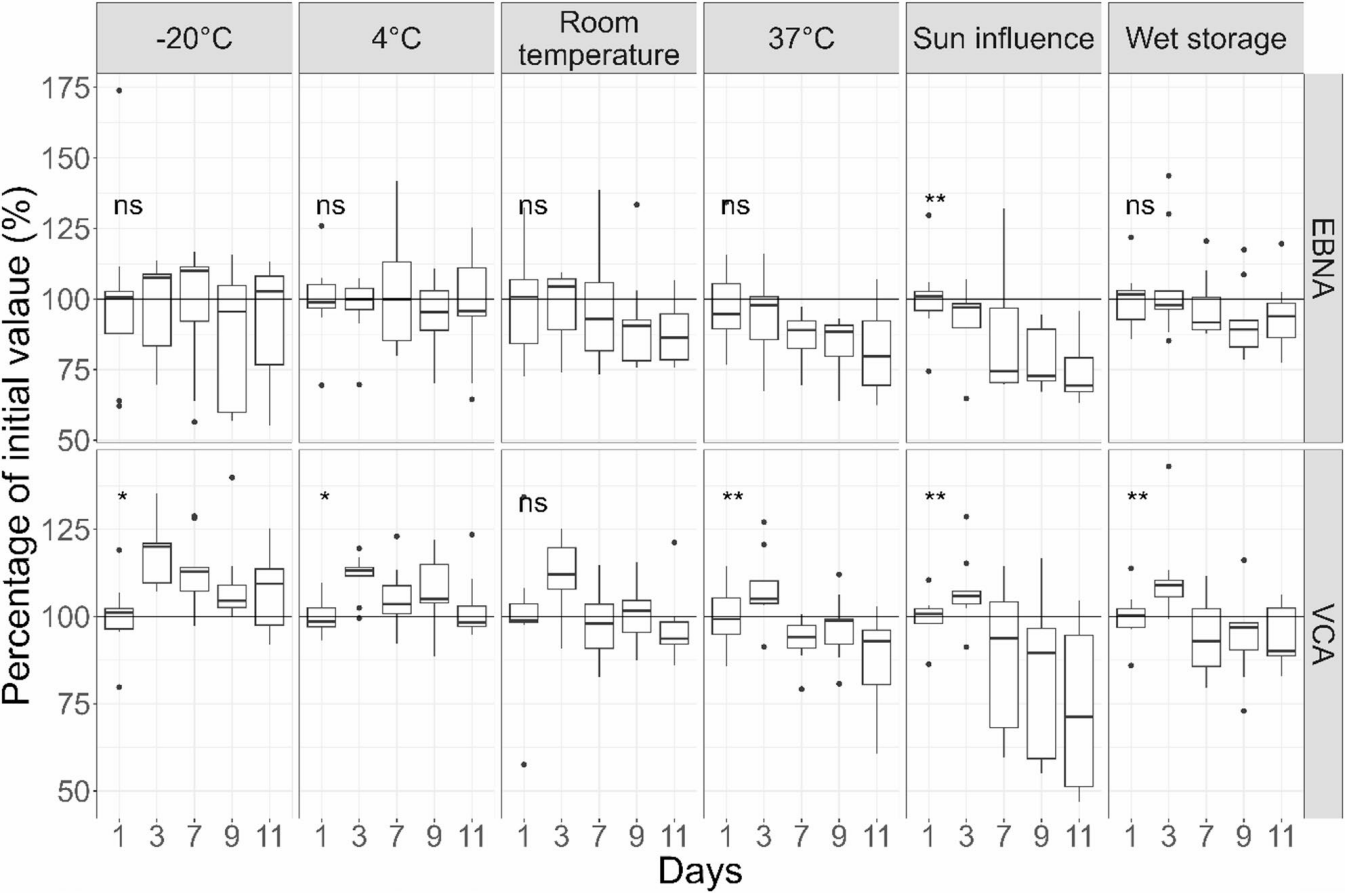
Decay characteristics of titres obtained from DBS-eluates from three different subjects, each tested in triplicate over time. Punching and analysis were performed on the same day at the indicated intervals. Chosen conditions: 4–7 C (4 : fridge condition): DBS were dried at room temperature, packed in zipper bags, and stored in a refrigerator; 36–38 C (37 : hot condition): DBS were dried at room temperature, packed, and stored in an incubator of aforementioned temperature. Before each re-insertion, DBS were re-packed to prevent condensation; Sun influence: DBS were dried for 12 h, packed, and then exposed to direct sunlight on a windowsill for one day. Subsequent storage was at room temperature. Wet storage: DBS were dried for only 3 h at room temperature in the shade and then sealed in damp status in zipper bags at room temperature for further storage without desiccant. They were removed briefly for punching and immediately reinserted. In this group, punching errors occurred due to the paper being floppy and wet. P-values are given by the Wilcoxon test: ns: *p* > 0.05, *: *p* ≤ 0.05, **: *p* ≤ 0.01

To evaluate whether improper storage of self-collected DBS cards during home drying or shipment could compromise sample integrity and affect results, the preanalytical stability of antibody titers was assessed under various conditions simulating real-life handling scenarios by laypersons or during transit (Fig. 5). Significant deterioration of anti-EBNA antibody levels was observed only in the sunlight-exposed group, with relevant declines beginning after day 3. Notably, in the anti-VCA measurements, all conditions revealed significant changes except for room temperature, showing only a trend towards slightly higher values around day 3. Interestingly, anti-VCA titers increased between days 3 and 7 across all conditions, subsequently returning to baseline; only the sunlight-exposed samples showed deterioration beyond baseline levels.

## Discussion

Establishing a reliable DBS-based testing strategy is particularly relevant given the high prevalence of EBV among adults and many subsequent morbidities that arise from EBV infections. Clinical trials investigating interventions, such as vaccines or medications, depend on identifying EBV-negative individuals who are relatively rare in adult populations. Large-scale screening efforts are therefore essential. DBS sampling is especially well-suited for this purpose: it is minimally invasive, cost-effective, does not require trained medical personnel, and allows for easy transport and long-term stability under various storage conditions [10].

In the past, McDade et al. [12] and Eick et al. [8], successfully implemented DBS-eluate based serology testing for EBV antibodies using an ELISA format. McDade et al. [12] validated a method for detecting anti-VCA antibodies in DBS and applied it in a large community-based study on child and adolescent psychopathology. They compared paired DBS and serum samples from 40 adults in a single assay, finding a strong linear correlation (Pearson *R* = 0.97) and establishing a DBS-specific seropositivity threshold. However, as the validated ELISA protocol for DBS has become unavailable, Eick et al. [8] also validated a commercial ELISA for detecting anti-VCA IgG antibodies in DBS by analyzing paired serum, fingerprick DBS, and venous blood DBS samples from 208 individuals aged 18–77 years. The assay showed strong linearity and minimal bias (-0.07 by Bland-Altman analysis), with a high correlation between serum and DBS titers (R² = 0.93). The study also assessed antibody stability under different storage conditions, confirming that DBS cards stored at room temperature, − 20 °C, and − 80 °C remained stable, whereas high temperatures (> 37 °C) reduced antibody levels.

In our study, we used 416 paired serum and DBS samples and the validated Roche Elecsys^®^ EBV EBNA IgG and Elecsys^®^ EBV VCA IgG assays. To our knowledge, this is the first time that a modern electro-luminescence based automated assay format was validated for anti-EBV serology using DBS-eluates. With a classification tree approach, we identified stable cutoffs for anti-EBNA antibodies (0.15 COI) and anti-VCA antibodies (2.3 COI), achieving an overall high sensitivity of 98.8% and a specificity of 96.5%. COI values were used as a semi-quantitative proxy of antibody levels without modifying the assay’s qualitative classification. Classification trees were chosen as method to minimize simultaneously false negatives/positive in both assays, increasing the sensitivity of the global method (both anti-EBNA and anti-VCA simultaneously) compared to separated sensitivities and specificities for each assay. A sequential method would evaluate potential cutoffs by minimizing the total misclassification error, treating each antibody independently.

Strong correlations between DBS and serum values were observed, in line with previous findings by Eick et al. [8], McDade et al. [12], and our previous studies [9, 10]. Moreover, predictive formulas similar to those in our previous study were derived, allowing the estimation of serum-equivalent units from DBS measurements with high precision. This enhances the utility of DBS in contexts where venous puncture-derived serum collection is impractical, with serum-equivalent units enabling the comparison of results obtained from DBS-based investigation and those from conventional serum-based analyses, thus supporting its application in large-scale sero-epidemiological studies and the comparison with previous studies performed with the Roche Elecsys EBV assays.

To assess sample stability, we examined anti-EBNA and anti-VCA antibody degradation under six different storage conditions over 11 consecutive days, covering the estimated timeframe expected for transportation and analysis. Anti-EBNA antibody levels showed significant deterioration only when stored in a hot incubator or exposed to direct sunlight, with a slight trend after day 7. Anti-VCA antibody levels were more sensitive to environmental conditions, showing an increase between days 3 and 7 and then significant declines back to baseline in almost all conditions for more than a week. Therefore, anti-VCA antibody values need to be considered carefully as the assay seems to be less robust than the more stable anti-EBNA measurements. Based on these findings, it is recommended to store DBS samples in a freezer/refrigerator and to avoid exposure to heat, sunlight, or moisture for prolonged times. Similarly to our previous study and Eick et al. [8] we observed deterioration of antibody stability at high temperatures (above 37 °C) relatively quickly. Additionally, in our previous studies we observed degradation due to sunlight exposure and wet packaging, in accordance with the findings of this study.

While other studies found limitations in the usage of self-sampled DBS [11] we observed no major differences either in the quality or in the value-distributions between DBS samples collected via self-sampling and those collected by medical personnel (Supplementary Figure S1), indicating comparability across collection methods. This is in alignment with our previous experiences [9, 23], and demonstrates the usefulness of our approach for unsupervised home-sampling, even though our two cohorts showed slightly different positivity rates, caused by different recruitment times and cohorts.

For anti-EBNA antibodies, the LOESS regression closely followed a linear trend, consistent with the linear regression results. In contrast, the LOESS curve for anti-VCA antibodies exhibited slight non-linearity, resembling a parabolic shape. Nevertheless, the linear approximation remained acceptable for analytical purposes.

In our study, 79.3% (330/416) of samples were either anti-VCA and/or anti-EBNA-positive. As the sample comprised individuals aged 18–25 years, primarily recruited in Munich through university settings, and right after the SARS-CoV-2 pandemic times with reduced intimate contacts among teenagers, the generalizability of the observed seroprevalence may be limited. Broader data collection including other populations could further solidify the observed calculation formula, although it is not expected to change significantly. In summary, our findings provide valuable insights for development of semi-quantitative anti-EBV electroluminescence assays for DBS-eluates, providing information for future vaccine studies and optimizing recruitment strategies in the field of EBV epidemiology.

## Conclusion

We derived a protocol for detecting EBV-specific antibodies against EBNA-1 and VCA, established for serum, for use in DBS. DBS-samples can easily be self-collected by participants at home and are more cost- and resource-efficient compared to conventional serum sampling. The established protocol was found to be suitable for high-throughput analysis and allows for the robust assessment of sero-status in a large population, independent of the collection method. In addition, given the high background EBV prevalence in the population, our approach supports easy and accurate pre-screening for the identification of EBV-negative participants for future clinical trials.

## Electronic Supplementary Material

Below is the link to the electronic supplementary material.

Supplemental Figure S1: Density plot showing the distribution of raw cut-off index (COI) values derived from DBS samples collected by medical personnel (n=416) (blue) and self-collected DBS samples (n=295) (grey) for (A) anti-EBNA antibodies and (B) anti-VCA antibodies. The dashed vertical lines indicate the cutoffs for classification (0.15 COI for anti-EBNA; 2.3 COI for anti-VCA).

## Data Availability

Our data are accessible to researchers upon reasonable request to the corresponding author taking data protection laws and privacy of study participants into account. To facilitate reproducibility and reuse, the analysis and figure generation code has been made available on Zenodo (https://zenodo.org/records/21333309).

## Abbreviations

BCa: Bias-corrected and accelerated
CI: Confidence interval
CLIA: Chemiluminescence immunoassay
COI: Cut-off Index
DBS: Dried blood spots
EBV: Epstein-Barr virus
EBNA-1: EBV nuclear antigen-1
ELISA: Enzyme-linked immunosorbent assay
ELECSYS: Electrochemiluminescence
HL: Hodgkin lymphoma
IVD: In vitro diagnostic
IM: Infectious mononucleosis
LOESS fits: Locally estimated scatterplot smoothing or local regression
MS: Multiple sclerosis
PIA: Prospective Moitoring and Management Application
VCA: Viral capsid antigens
