# Supplementary figures and images for: A dried blood spot protocol for high-throughput semi-quantitative analysis of Epstein-Barr Virus VCA IgG and EBNA IgG serologies based on the Roche Elecsys system
Source: Virol J. 2026 Jul 23;23:178. doi: 10.1186/s12985-026-03219-w (PMC13401283; doi:10.1186/s12985-026-03219-w)

A

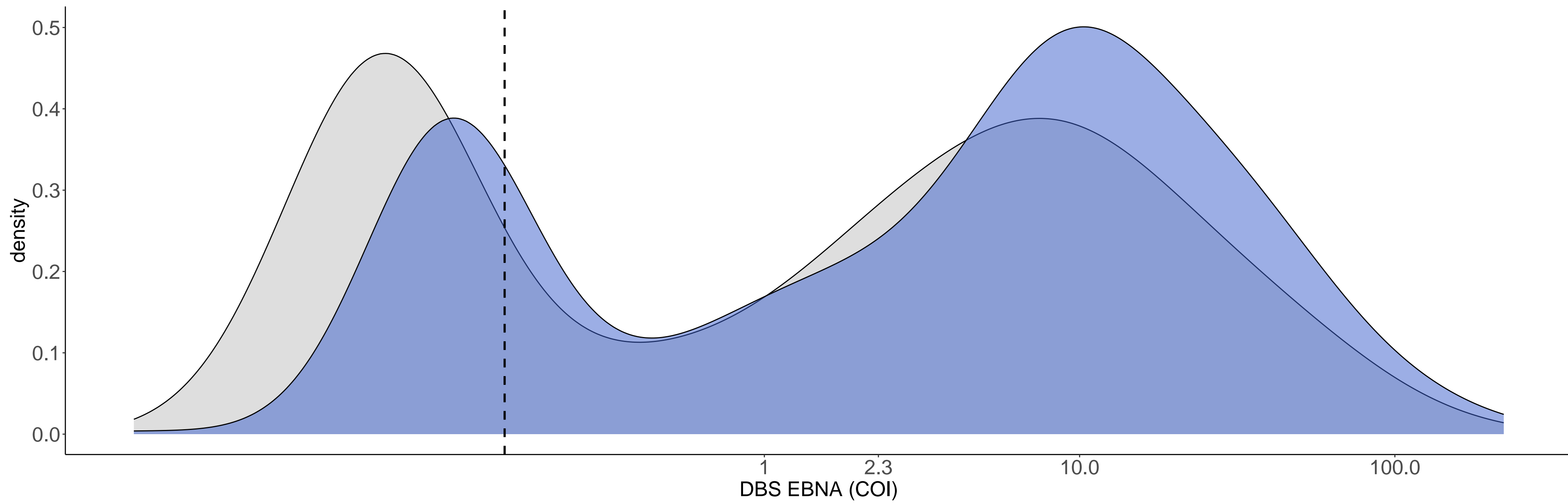

B

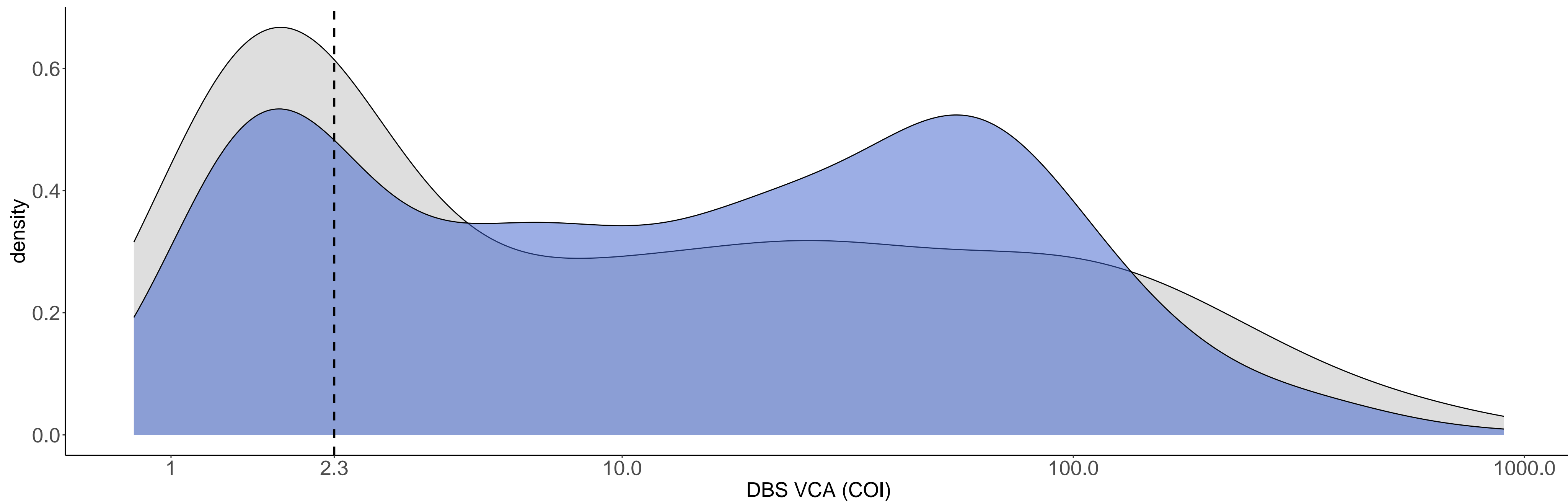

Supplement: Supplementary file 1 — Supplemental Figure S1: Density plot showing the distribution of raw cut-off index (COI) values derived from DBS samples collected by medical personnel (n=416) (blue) and self-collected DBS samples (n=295) (grey) for (A) anti-EBNA antibodies and (B) anti-VCA antibodies. The dashed vertical lines indicate the cutoffs for classification (0.15 COI for anti-EBNA; 2.3 COI for anti-VCA). [file 12985_2026_3219_MOESM1_ESM.pdf]
